# Supplementary material for: MetaRibo-Seq measures translation in microbiomes
Source: Nat Commun. 2020 Jun 29;11:3268. doi: 10.1038/s41467-020-17081-z (PMC7324362; doi:10.1038/s41467-020-17081-z)
Supplement: Supplementary file 10 — Supplementary Data 7 [file 41467_2020_17081_MOESM10_ESM.zip › File2/Confidence_High_Taxonomy/369926_out.krona.html]

Javascript must be enabled to view this page.

members
magnitude
magnitudeUnassigned
count
unassigned
taxon
rank

369926\_out

3

2
superkingdom
3

phylum
3
976

3
class
200643

171549
3
order

2005525
3
family

genus
3
375288

46503
species
3

SRS016056\_contig\_number\_2593SRS077552\_contig\_number\_28609SRS143991\_contig\_number\_21528
